# Fully automated coronary artery calcium quantification on electrocardiogram-gated non-contrast cardiac computed tomography using deep-learning with novel Heart-labelling method

**DOI:** 10.1093/ehjopen/oead113

**Published:** 2023-11-08

**Authors:** Daigo Takahashi, Shinichiro Fujimoto, Yui O Nozaki, Ayako Kudo, Yuko O Kawaguchi, Kazuhisa Takamura, Makoto Hiki, Eisuke Sato, Nobuo Tomizawa, Hiroyuki Daida, Tohru Minamino

**Affiliations:** Department of Cardiovascular Biology and Medicine, Juntendo University Graduate School of Medicine, 2-1-1 Hongo Bunkyo-ku, Tokyo 113-8421, Japan; Department of Cardiovascular Biology and Medicine, Juntendo University Graduate School of Medicine, 2-1-1 Hongo Bunkyo-ku, Tokyo 113-8421, Japan; Department of Cardiovascular Biology and Medicine, Juntendo University Graduate School of Medicine, 2-1-1 Hongo Bunkyo-ku, Tokyo 113-8421, Japan; Department of Cardiovascular Biology and Medicine, Juntendo University Graduate School of Medicine, 2-1-1 Hongo Bunkyo-ku, Tokyo 113-8421, Japan; Department of Cardiovascular Biology and Medicine, Juntendo University Graduate School of Medicine, 2-1-1 Hongo Bunkyo-ku, Tokyo 113-8421, Japan; Department of Cardiovascular Biology and Medicine, Juntendo University Graduate School of Medicine, 2-1-1 Hongo Bunkyo-ku, Tokyo 113-8421, Japan; Department of Cardiovascular Biology and Medicine, Juntendo University Graduate School of Medicine, 2-1-1 Hongo Bunkyo-ku, Tokyo 113-8421, Japan; Department of Radiological Technology, Faculty of Health Science, Juntendo University, 2-1-1 Hongo Bunkyo-ku, Tokyo 113-8421, Japan; Department of Radiology, Juntendo University Graduate School of Medicine, 2-1-1 Hongo Bunkyo-ku, Tokyo 113-8421, Japan; Department of Cardiovascular Biology and Medicine, Juntendo University Graduate School of Medicine, 2-1-1 Hongo Bunkyo-ku, Tokyo 113-8421, Japan; Department of Radiological Technology, Faculty of Health Science, Juntendo University, 2-1-1 Hongo Bunkyo-ku, Tokyo 113-8421, Japan; Department of Cardiovascular Biology and Medicine, Juntendo University Graduate School of Medicine, 2-1-1 Hongo Bunkyo-ku, Tokyo 113-8421, Japan

**Keywords:** Coronary artery calcium, Computed tomography, Artificial intelligence, Deep learning

## Abstract

**Aims:**

To develop an artificial intelligence (AI)-model which enables fully automated accurate quantification of coronary artery calcium (CAC), using deep learning (DL) on electrocardiogram (ECG)-gated non-contrast cardiac computed tomography (gated CCT) images.

**Methods and results:**

Retrospectively, 560 gated CCT images (including 60 synthetic images) performed at our institution were used to train AI-model, which can automatically divide heart region into five areas belonging to left main (LM), left anterior descending (LAD), circumflex (LCX), right coronary artery (RCA), and another. Total and vessel-specific CAC score (CACS) in each scan were manually evaluated. AI-model was trained with novel Heart-labelling method *via* DL according to the manual-derived results. Then, another 409 gated CCT images obtained in our institution were used for model validation. The performance of present AI-model was tested using another external cohort of 400 gated CCT images of Stanford Center for Artificial Intelligence of Medical Imaging by comparing with the ground truth. The overall accuracy of the AI-model for total CACS classification was excellent with Cohen’s kappa of *k* = 0.89 and 0.95 (validation and test, respectively), which surpasses previous research of *k* = 0.89. Bland-Altman analysis showed little difference in individual total and vessel-specific CACS between AI-derived CACS and ground truth in test cohort (mean difference [95% confidence interval] were 1.5 [−42.6, 45.6], −1.5 [−100.5, 97.5], 6.6 [−60.2, 73.5], 0.96 [−59.2, 61.1], and 7.6 [−134.1, 149.2] for LM, LAD, LCX, RCA, and total CACS, respectively).

**Conclusion:**

Present Heart-labelling method provides a further improvement in fully automated, total, and vessel-specific CAC quantification on gated CCT.

## Graphical Abstract

**Graphical abstract:**
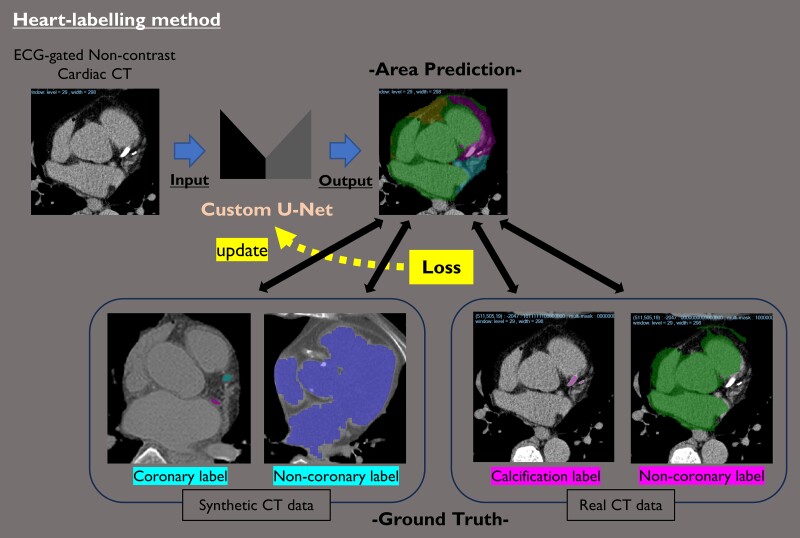

## Introduction

Coronary artery calcium (CAC) score (CACS) is widely used for risk assessment of coronary artery disease (CAD)^1^ and has been integrated into several clinical guidelines. Numerous previous studies have demonstrated that the conventional quantitative evaluation by CAC scoring on electrocardiogram (ECG)-gated non-contrast cardiac computed tomography (gated CCT) serves a great value of predicting CAD.^2,3^ Recent research has also revealed the prognostic impact bestowed by branch-wise CAC assessment.^4–6^ However, to calculate CACS in every single patient for CAD screening is too inefficient because it requires experts’ manual identification of coronary calcium lesions.

On the other hand, in recent years, artificial intelligence (AI) technologies have made great strides and demonstrated their efficacy in several medical fields. Especially in medical diagnostic imaging, deep learning has a big potential to assist in detecting lesions or abnormalities.^7–9^ Although some software has already been developed to quantify total CACS full-automatically,^10,11^ identification of localized calcification on each coronary vessel is still challenging. The aim of this study is to develop a further accuracy-improved AI-model for both total and vessel-specific CAC scoring on gated CCT. The key contributions of our study are:

- Explainable AI-model for Agatston scoring; performs heart labelling into coronary artery perfusion regions followed by well-proven rule-based calcium detection with CT thresholding of 130 Hounsfield units (HU) rather than black box approach of direct calcification detection
- Diverse training and validation cohorts, also containing normal CCTs without calcification
- Test on external public dataset with accuracy better than state of the art.

## Methods

This retrospective study was approved by our institutional review board in accordance with local ethics procedures, and the requirement for written informed consent was waived.

### Study design and population

Three independent cohorts were studied, including a total of 1369 patients who underwent gated CCT.

#### Training cohort

**Figure 1:**
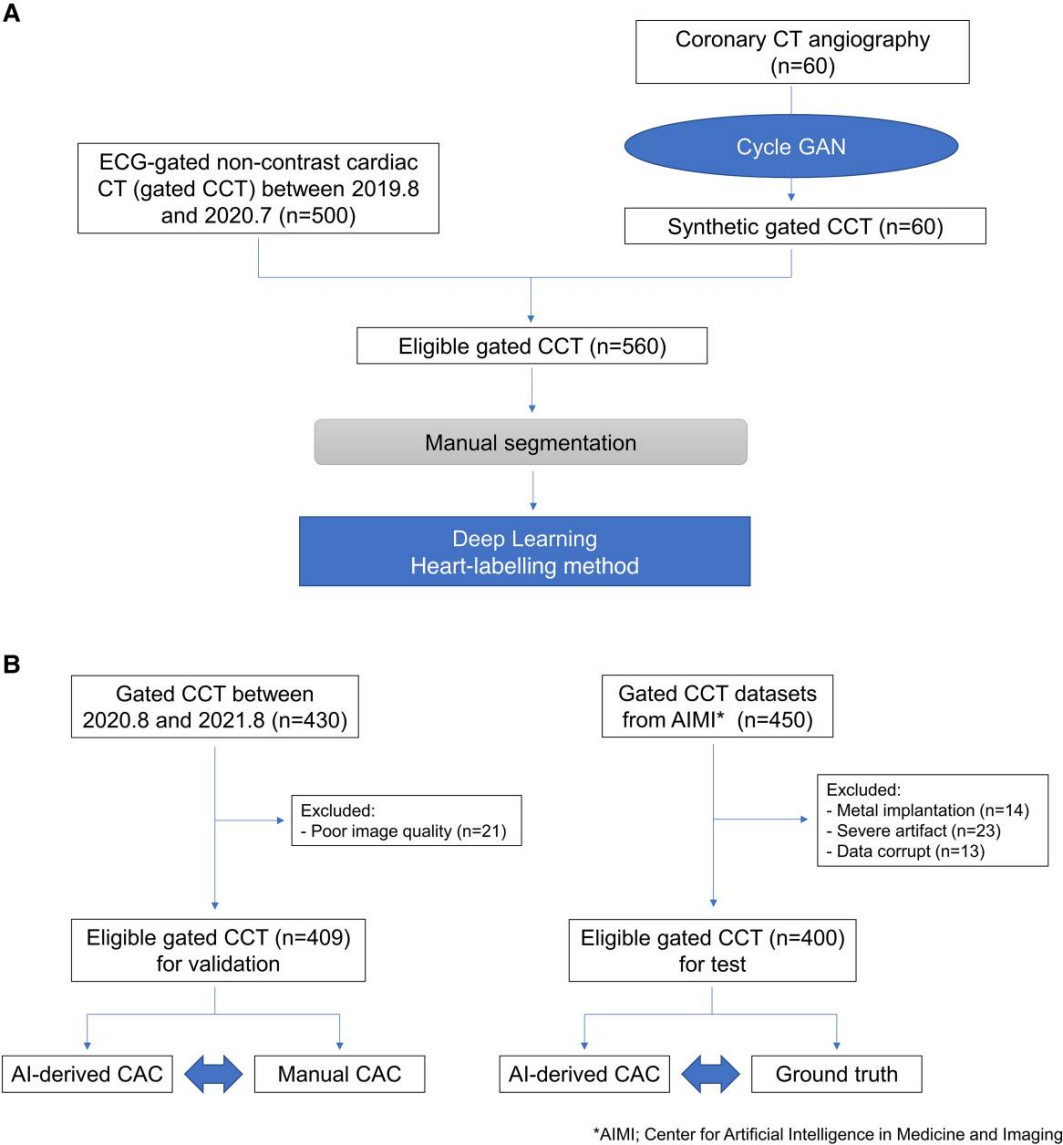
(*A*) Study flow chart of training cohort. A total of 560 gated CCT images were used for model training, including 60 synthetic gated CCT generated from CCTA images. (*B*) Study flow chart of validation and test cohort. For validation, as 21 gated CCT images were excluded due to its poor image quality or artefacts, a total of 409 gated CCT images were enrolled. For test, 50 scans were excluded because of several reasons, and a total of 400 gated CCT images were used. The performance of the model was assessed by comparing AI-derived CACS with manual CACS, or ground truth segmentation (validation cohort and test cohort, respectively) in both total and vessel-specific levels.

Study flow chart model training is shown in *Figure 1A*. We retrospectively studied 500 consecutive patients who had no prior history of heart disease and underwent coronary CT angiography (CCTA) for suspected of CAD in our institution between August 2019 and July 2020. In every patient, gated CCT scans were also obtained just before CCTA for routine CAC scoring. An additional 60 synthetic gated CCT images were generated from the other CCTA cases (detailed later); therefore, a total of 560 gated CCT images were used for model training.

#### Validation and test cohort

Study flow chart of model validation and test is shown in *Figure 1B*. For performance validation of the AI-model, 430 consecutive patients who had no prior history of heart disease and underwent gated CCT and CCTA for clinical indication for suspected CAD in our institution between August 2020 and August 2021 were enrolled. Twenty-one scans were excluded due to its poor image quality or artefacts; thus, a total of 409 gated CCT images were used for model validation. The performance of the model was assessed by comparing AI-derived CACS with manually-derived CACS in both total and vessel-specific levels. The validation cohort played an integral role in monitoring and fine-tuning the model during training to avoid overfitting.

For performance testing of the AI-model, we studied another 450 gated CCT datasets that Stanford University has made available online for free (Center for Artificial Intelligence in Medicine and Imaging; AIMI).^12^ To be used for CACS analysis, these datasets consist of the images obtained from the patients with at least some calcification in their coronary arteries. Fifty scans were excluded because of metal implants, severe artefacts, or data corruption (14, 23, and 13, respectively); then, a total of 400 gated CCT images were enrolled. We compared AI-derived CACS with the disclosed ground truth segmentation. The test cohort was employed only for evaluation purposes, and at no point was it used to influence or impact the training process.

### Imaging protocol

All the patients in the internal training and validation cohorts were scanned using a 320-row CT (Aquillion ONE, Canon medical systems, Tokyo, Japan). Prospective gated CCT was obtained just before CCTA, at 75% of R-R interval, with a tube current of 50–250 mA (depending on body mass index), and voltage of 120 KVp. The slice thickness was 3.0 mm. Images of the external test cohort were obtained with a same voltage and slice thickness; however, the other detailed conditions are not disclosed.

### Manual coronary artery calcium detection, calculation, and categorization

Vessel-specific CAC identification was performed by an expert on an axial view of gated CCT images, and each calcification was labelled using one of the four labels [left main (LM), left anterior descending (LAD), left circumflex (LCX), and right coronary artery (RCA)] using dedicated software (SYNAPSE VINCENT, Fujifilm, Japan). Complementary information from sagittal and coronal views was also used as a reference. As described in conventional studies, the threshold for a calcific lesion was set at CT value of 130 HU, having area ≥ 1 mm^2^.^13,14^ Then CACS was calculated using the method of Agatston,^3^ and the scores were categorized into five risk groups as previously described: (0) <1, (1) 1–10, (2) 11–100, (3) 101–400, and (4) >400.^15,16^ Patients in test cohort had at least a little coronary calcification; thus they were stratified into four latter groups. In training cohort, second manual CAC segmentation was performed 3 months after the initial analysis and also by another expert to assess intra- and inter-observer agreement to certify reproducibility of CAC quantification.

### Artificial intelligence algorithm methodology

#### Heart-labelling method

**Figure 2:**
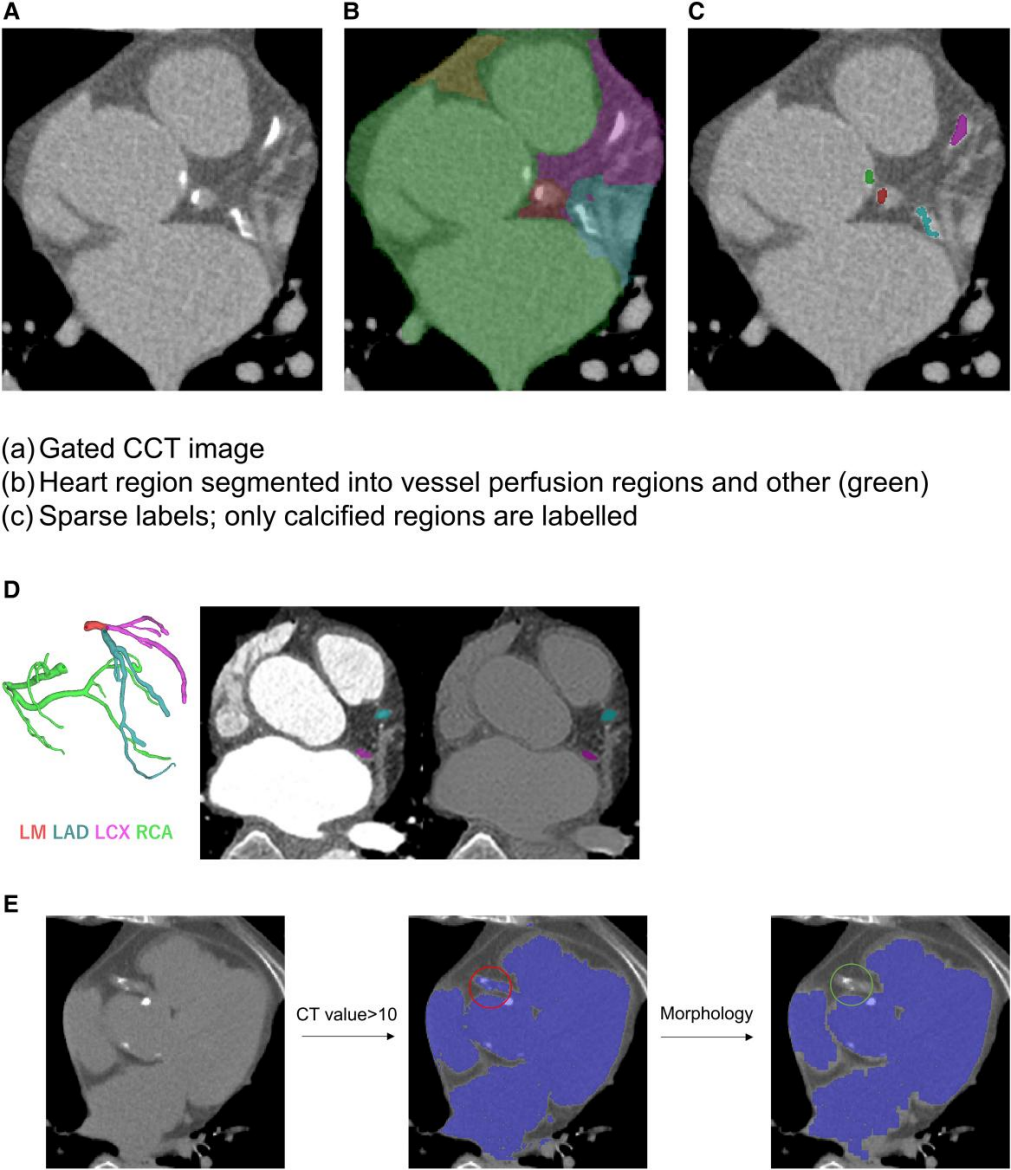
Heart-labelling method. (*A*) Gated CCT image. Calcifications are localized in LM, LAD, LCX and another (aorta) region. (*B*) Heart labelling into vessel perfusion regions and other region (green). (*C*) Sparse labels; only calcified regions are labelled. (*D*) Coronary labels. In 3D coronary CT angiography, whole coronary arteries are labelled (left). The synthetic image is generated by transforming contrast enhanced image (mid) into non-contrast image (right). (*E*) Non-coronary labels. Non-coronary label generation. Original CCT image (left), labelling by CT value threshold (middle), removal of the coronary artery region by applying multiple erosion operations followed by dilation which removes any thin tube-like structures (right).

Conventionally, AI-based CAC scoring algorithms detect calcifications on gated CCT images directly.^10,11^ An ideal and explainable approach would be to extract and label coronary arteries in CCT images using the AI, followed by calcium extraction using threshold of 130 HU. However, the extraction of coronary arteries is challenging due to poor visibility in thick slice scan (3 mm). Thus, we propose a more practical yet explainable approach of extracting vessel perfusion regions by segmenting CCT images into five regions; four perfusion regions of LM, LAD, LCX, and RCA and another (e.g. aorta, valves, ventricles; the other area or structures inside the mediastinum.). *Figure 2A* shows gated CCT image, and *Figure 2B* shows heart segmented into five regions. We refer to this method as ‘Heart-labelling method’.

#### Heart-labelling from sparse labels

Only calcified regions are labelled in the ground truth as shown in *Figure 2C*. From the context of Heart-labelling into perfusion regions, these labels are sparse labels; only a part of perfusion region is labelled. In the field of DL, training using such sparse labels is conducted by masking out unlabelled regions while training.^17^ Masking out means AI is trained to correctly output only the labelled regions, while the output at unlabelled regions is ignored. The hypothesis is that, if we consider the entire dataset, the labelled calcified regions are distributed uniformly across various regions in the perfusion area, allowing the AI to learn underlying perfusion area.

#### Additional anatomical labels for better accuracy

The assumption that calcified regions are representative of the entire perfusion region is not always true and requires very large datasets. Because regions that fall outside of the defined calcified labels would typically be disregarded, allowing the AI to attribute potentially arbitrary probabilities to those uncertain areas, the use of sparse labels alone might produce noisy labels. So, to further guide the AI to learn the underlying perfusion area, we create additional anatomical labels.

First, we create a small dataset in which entire coronary arteries are labelled. From 60 scans of CCTA, synthetic gated CCT images were generated using CycleGAN.^18^ Our implementation is derived from a recently proposed non-contrast to contrast image translation using CycleGAN.^19^ The coronary arteries clearly visible in CCTA were manually labelled and transferred to CCT images as shown in *Figure 2D*.

Second, to guide the AI to recognize the non-coronary region, also referred to as other region in this work, we automatically generate the labels for it. The labels are generated automatically using simple thresholding and morphology operations. As shown in *Figure 2E*, within the heart region, the areas with CT value greater than 10 HU are extracted. To remove coronary artery region, morphology operations of multiple erosions followed by dilation were applied so that it can remove any tube-like thin structures.

#### Convolutional neural network

**Figure 3:**
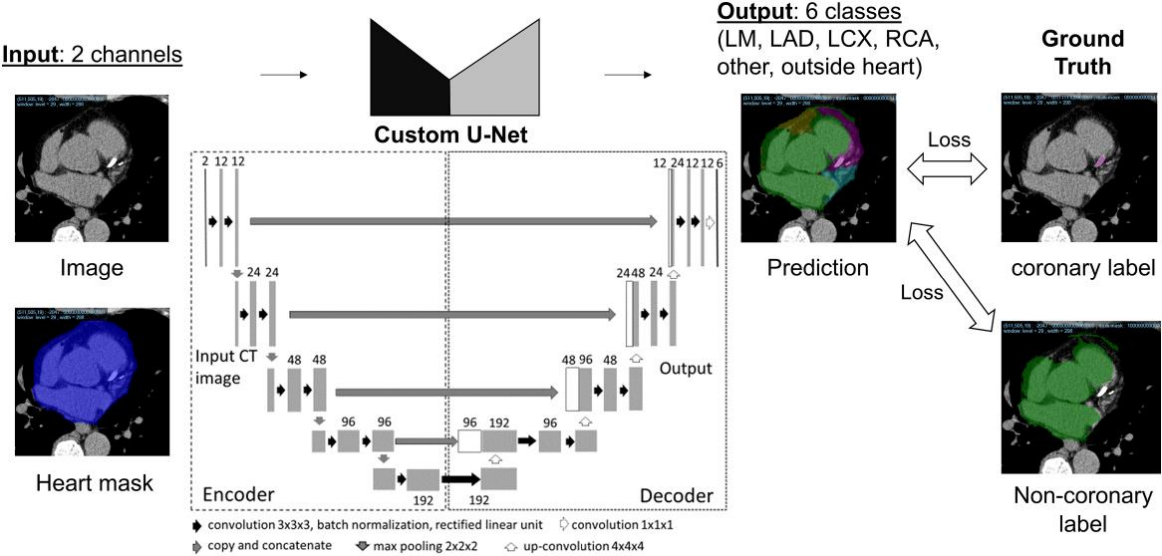
AI-algorithm using U-Net architecture. AI-algorithm consists of two channel input of a plain CCT image and a heart mask, and six channel outputs of LM, LAD, LCX, RCA, another, and outside heart classes. Three-dimensional fully convolutional neural network consists of an encoder and a decoder, which have convolutional filters, and the weights of the convolution layers are updated point by point so that the loss is minimized.

*Figure 3* shows a training flow using 3D fully convolutional neural network (FCNN) derived from U-Net architecture,^20^ which was trained to segment the heart into five regions: LMT, LAD, LCX, RCA, and another. It takes two input channels, 3D CT volume and binary mask of heart region, and six output channels, comprised of probability maps corresponding to LMT, LAD, LCX, RCA, another, and outside heart classes. Three-dimensional FCNN consists of an encoder and a decoder, each of which consists of several convolutional filters through which the image is processed to produce the output. The weights of the convolution filters are updated during training so that the loss (computed as difference between AI output and ground truth) is minimized. In this work, we use multi-class dice loss function. To make the AI robust to various scanning protocols (resolution, noise levels, position), we apply data augmentation during the training. The details of loss function and data augmentation are provided in Supplementary material online, File  *S1*.

### Statistical analysis

All statistical analysis was performed using JMP 14.2 (SAS Institute Inc., Cary, NC, USA). For the training cohort, intra- and inter-observer variabilities were assessed using Pearson’s correlation coefficient and Bland–Altman (B-A) analysis. For validation and test cohorts, the risk category performance of the AI-model was assessed by the sensitivity, specificity, positive predictive value (PPV), negative predictive value (NPV), accuracy, and kappa value. The concordance of total and vessel-specific CACS between AI prediction and ground truth were assessed by B-A plot and correlation metrics using the method of Pearson. Kolmogorov–Smirnov (K-S) test was also used to analyse the similarity of distributions of AI-derived CACS and ground truth. Normally distributed quantitative variables are presented with mean and 95% CI. Non-normally distributed data was presented with median and interquartile range (IQR). A statistically significant difference was defined as a two-sided *P*-value <0.05.

## Results

### Baseline characteristics

Characteristics of all patients included in training and validation cohort are shown in *Table 1*. The mean age was 63.5 ± 13.3 and 65.8 ± 11.7 years, and 38.8% and 43.8% were female. The mean body mass index was 23.4 ± 3.7 and 23.5 ± 3.3. The median CACS was 32.6 and 22.8, respectively.

**Table 1:** Baseline characteristics of the training and validation cohort

	Training cohort (*n* = 500)	Validation cohort (*n* = 409)
Age (years)	63.5 ± 13.3	65.8 ± 11.7
Sex		
Female, *n* (%)	194 (38.8)	179 (43.8)
Male, *n* (%)	306 (61.2)	230 (56.2)
Body mass index (kg/m^2^)	23.4 ± 3.7	23.5 ± 3.3
Risk factor		
Hypertension, *n* (%)	263 (52.6)	235 (58.4)
Dyslipidemia, *n* (%)	265 (53.0)	202 (49.4)
Diabetes mellitus, *n* (%)	144 (28.8)	118 (28.9)
Family history of CAD, *n* (%)	132 (26.4)	80 (19.6)
Current smoking, *n* (%)	71 (14.2)	71 (17.4)
Blood test		
LDL-Cholesterol (mg/dL)	109.9 ± 32.2	112.5 ± 49.9
HDL-Cholesterol (mg/dL)	56.1 ± 15.9	58.2 ± 17.3
Triglyceride (mg/dL)	137.2 ± 93.6	143.9 ± 124.1
Hemoglobin A1c (%)	6.3 ± 1.1	6.2 ± 1.1
Serum creatinine (mg/dL)	0.78 [0.65, 0.87]	0.77 [0.64, 0.89]
Agatston score		
Total	32.6 [0, 200.9]	22.8 [0, 205.4]
LM	0 [0, 0]	0 [0, 0]
LAD	10.1 [0, 99.0]	5.7 [0, 97.5]
LCX	0 [0, 12.1]	0 [0, 12.3]
RCA	0 [0, 33.2]	0 [0, 33.3]

CAD, coronary artery disease; HDL, high density lipoprotein; LAD, left anterior descending; LCX, left circumflex; LDL, low density lipoprotein; LM, left main; RCA, right coronary artery

### Intra- and inter-observer reproducibility

**Figure 4:**
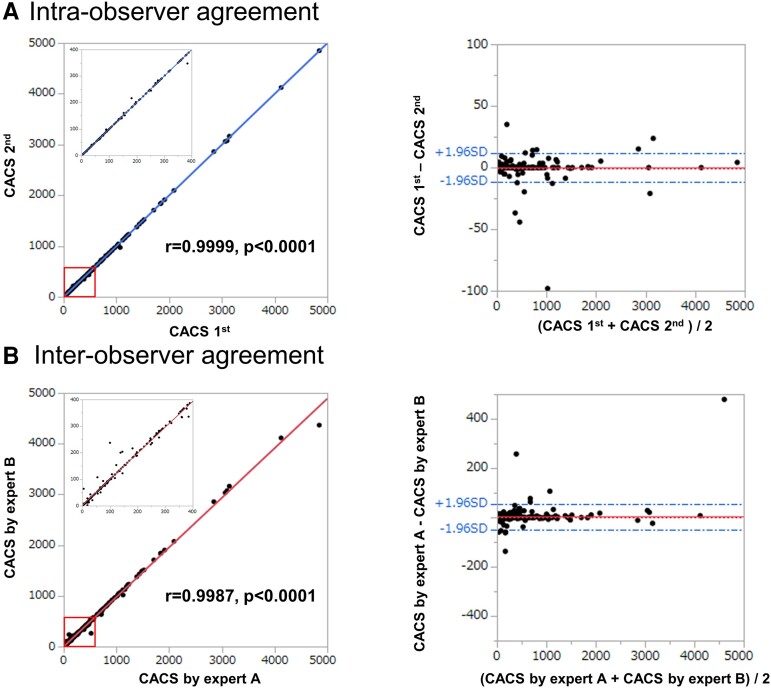
Reproducibility of CAC scoring. (*A*) Intra-observer agreement in total CACS quantification. Correlation plot between first analysis (*x*-axis) and second analysis (*y*-axis). The Pearson’s correlation coefficient was r = 0.9999. Also shown is an inset with a magnified view of scores in the range 0–400. In Bland–Altman plot, red dashed line shows mean difference (−0.2) with the two blue dashed lines representing upper and lower limit of agreement (LoA) of ±1.96SD ([−11.9, 11,4]). (*B*) Inter-observer agreement in total CACS quantification. Correlation plot between analysis by expert A (*x*-axis) and B (*y*-axis). The Pearson’s correlation coefficient was r = 0.9987. Also shown is an inset with a magnified view of scores in the range 0–400. In B-A plot, red dashed line shows mean difference (2.1) with the two blue dashed lines representing upper and lower limit of agreement (LoA) of ±1.96SD [(−51.2, 55.4).

*Figure 4A* and *4B* represent intra- and inter-observer agreement of CACS in training cohort, and we found excellent correlations (r = 0.9999, *P* < 0.0001; r = 0.9987, *P* < 0.0001, respectively). The graphical illustration in the form of B-A plots was also shown.

### Categorical assessment of total coronary artery calcium scoring

*Table 2a* represents that the overall accuracy of the AI prediction in the internal validation cohort. Total CACS classification was excellent with Cohen’s kappa of *k* = 0.89. The classification accuracy was 91.7% (375/409), with 24 false-positive cases. The sensitivity, specificity, PPV, and NPV were 100%, 82.7%, 91.8%, and 100%, respectively.

**Table 2:** Confusion matrix of CAC risk category

(a) Validation cohort						
	AI prediction					
Ground truth	Risk category	[0]	[1]	[2]	[3]	[4]
	[0]	115	15	9	0	0
	[1]	0	38	5	0	0
	[2]	0	0	84	2	0
	[3]	0	0	1	71	2
	[4]	0	0	0	0	67

(b) Test cohort						
	AI prediction					
Ground truth	Risk category	[0]	[1]	[2]	[3]	[4]
	[0]	N/A	N/A	N/A	N/A	N/A
	[1]	N/A	57	5	1	0
	[2]	N/A	0	126	4	1
	[3]	N/A	0	1	92	2
	[4]	N/A	0	0	0	111

Risk category: CAC score belongs to; [0], <1; [1], 1–10; [2], 10–100; [3], 100–400; [4], >400.

AI, artificial intelligence; CAC, coronary artery calcium.

Similarly, results of AI prediction in external test cohort can be found in *Table 2b*. Since patients without any CAC are not enrolled in this cohort, total CACS is categorized into only four groups [(1) 1–10, (2) 10–100, (3) 100–400, and (4) >400]. The accuracy was still excellent [96.5% (386/400)] with Cohen’s kappa of *k* = 0.95.

### Accuracy in total and vessel-specific coronary artery calcium scoring

**Figure 5:**
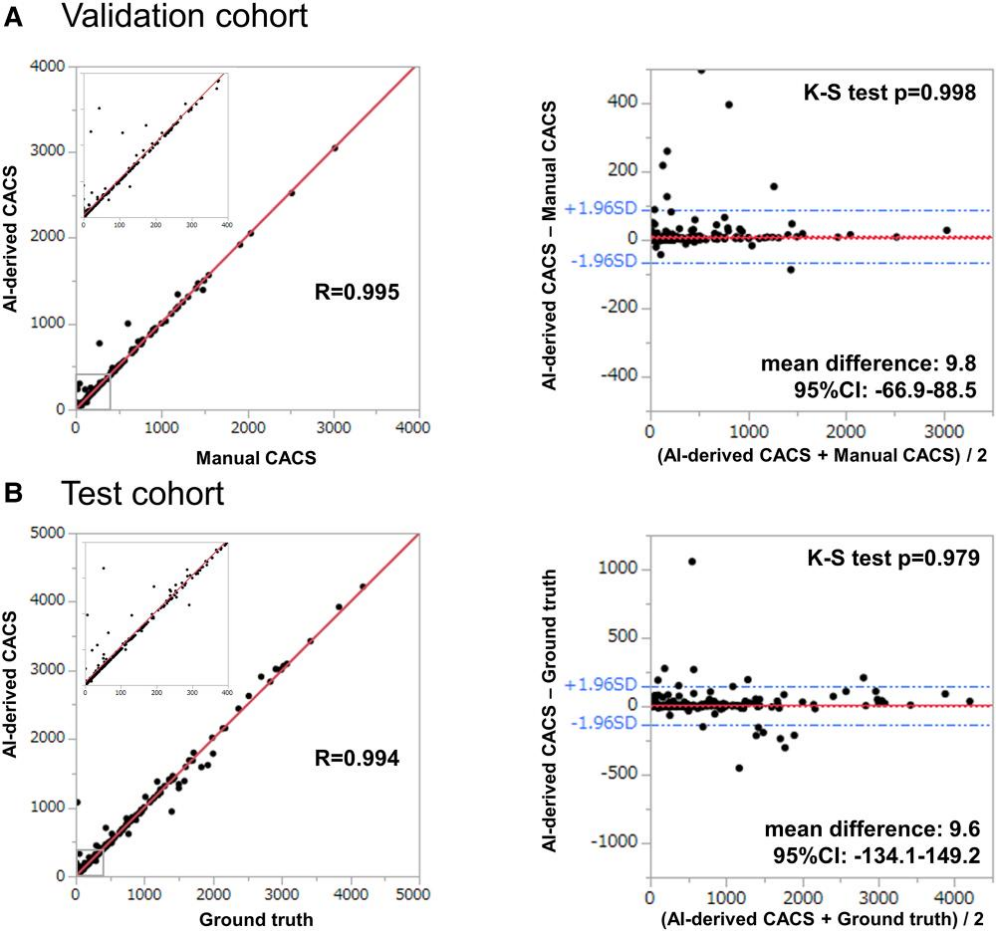
Performance of AI-derived total CAC scoring. For total CACS prediction, there was excellent correlation between AI-derived CACS and manual CACS (ground truth), in both validation and test cohort (r = 0.995, *P* < 0.0001; r = 0.994, *P* < 0.0001, respectively). B-A plots are also shown, suggesting little difference between two methods (mean difference [95%CI (confidence interval)]: 9.8 [−66.9, 88.5] and 9.6 [−134.1, 149.2], respectively). Kolmogorov-Smirnov (K-S) test showed no statistically significant difference in the distribution of CACS between two methods (*P* = 0.998 and 0.979, respectively).

**Figure 6:**
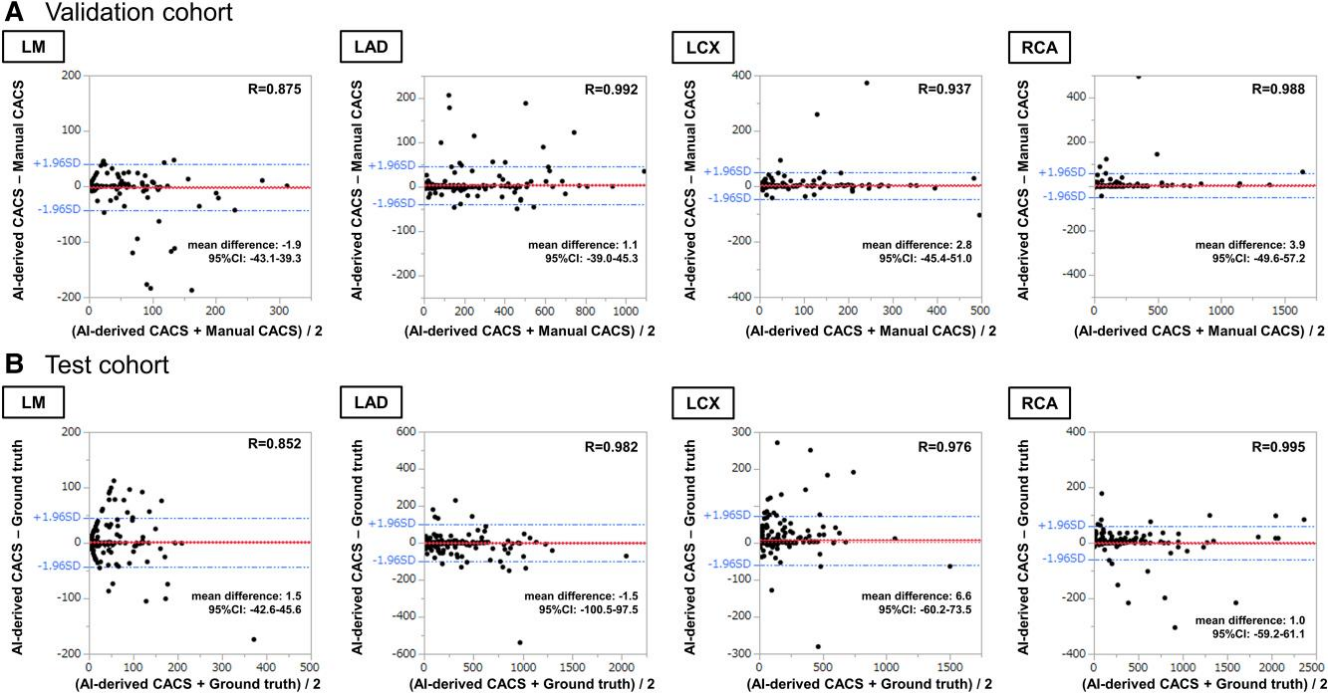
Performance of AI-derived vessel-specific CAC scoring. In validation cohort, AI-derived CACS excellently correlated with manual CACS (r = 0.875, 0.992, 0.937, and 0.988 for LM, LAD, LCX, and RCA, respectively), and B-A plots graphically represented little difference between two methods in vessel level (mean difference [95%CI] were −1.9 [−43.1, 39.3], 1.1 [−39.0, 45.3], 2.8 [−45.4–51.0], and 3.9 [−49.6, 57.2], in the same order). Correspondingly, excellent correlation and little difference were shown in test cohort (r = 0.852, 0.982, 0.976, and 0.995; mean difference [95%CI] were 1.5 [−42.6, 45.6], −1.5 [−100.5, 97.5], 6.6 [−60.2, 73.5], and 1.0 [−59.2, 61.1] for LM, LAD, LCX, and RCA, respectively).

*Figure 5* represents the comparison of the total CACS between AI-derived score and ground truth that was performed. We found excellent correlation between two methods, in both validation and test cohort (r = 0.995, *P* < 0.0001; r = 0.994, *P* < 0.0001, respectively). Bland–Altman plots are also shown, suggesting little difference between two methods {mean difference [95%CI (confidence interval)]: 9.8 [−66.9, 88.5] and 9.6 [−134.1, 149.2], respectively}. Kolmogorov–Smirnov (K-S) test showed no statistically significant difference in the distribution of CACS between two methods (*P* = 0.998 and 0.979, respectively). Vessel-specific results are shown in *Figure 6*. In validation cohort, AI-derived CACS excellently correlated with manual CACS (r = 0.875, 0.992, 0.937, and 0.988 for LM, LAD, LCX, and RCA, respectively), and B-A plots graphically represented little difference between two methods in vessel level [mean difference (95%CI) were −1.9 (−43.1, 39.3), 1.1 (−39.0, 45.3), 2.8 (−45.4–51.0), and 3.9 (−49.6, 57.2), in the same order]. Correspondingly, excellent correlation and little difference were shown in test cohort (r = 0.852, 0.982, 0.976, and 0.995; mean difference [95%CI] were 1.5 [−42.6, 45.6], −1.5 [−100.5, 97.5], 6.6 [−60.2, 73.5], and 1.0 [−59.2, 61.1] for LM, LAD, LCX, and RCA, respectively).

## Discussion

In present study, we have developed an improved comprehensive AI-model for automated CAC scoring on gated CCT at total and vessel-specific levels. Although it is theoretically possible to develop AI-models to calculate other indices of CAC (e.g. volume score, mass score), the present study focused on the Agatston score, which is the simplest, most widely used, and evidence-rich assessment method. By adopting a novel Heart-labelling method, AI-derived CAC scoring has achieved the following outcomes (compared to manual scoring): (i) excellent agreement in classification of CACS risk categories and (ii) excellent consistency in total and vessel-specific absolute CACS.

Fully automated CACS risk category classification has been validated in several research so far.^21,22^ In this study, our model achieved excellent accuracy in CACS classification with Cohen’s kappa *k* = 0.89 and 0.95 (validation and test cohorts, respectively). One recent research has demonstrated the great agreement in automated CACS classification of 79 patients with Cohen’s kappa *k* = 0.89.^23^ In the present model, a relatively large number of patients were analysed (*n* = 409 and 400), still maintaining or surpassing the previous reported model. In the validation cohort, there were 24 false positives which impaired overall result. Greater accuracy was achieved in the test cohort probably because it did not contain the case with CAC = 0.

Similarly, when treated as a continuous value, excellent CACS consistency was achieved between AI prediction and ground truth in both validation and test cohort (=0.993, *P* < 0.0001; r = 0.903, *P* < 0.0001, respectively). The reason why the latter one was lower is probably because the validation cohort includes 139 (33.9%) CAC-absent patients and 115 out of 139 CAC-absent cases (82.7%) were completely correctly analysed (as CAC = 0), which contributed to improving the overall accuracy. The mean differences were 9.8 and 9.6, which means AI prediction have tendency of slight overestimation.

Moreover, the incremental value of evaluating vessel-specific CAC has been demonstrated in several reports. It has been revealed that the presence and high burden of left main CAC are independently associated with greater cardiovascular and total mortality,^4,5^ and that the CAC of RCA is significantly associated with coronary heart disease.^6^ Another research has showcased that the presence of CAC in the proximal dominant coronary artery is associated with major coronary heart disease events (hazard ratio: 2.59).^24^ From this point of view, it can be said that vessel-specific CAC evaluation enables further risk assessment in addition to routine CAC scoring.

However, fully automated accurate CAC quantification of each coronary vessel has been a task with room for improvement, generally because of following problems: (i) misclassification between LM, LAD, and LCX, (ii) false positives from the calcification at aortic root and annulus, and (iii) false positives from non-coronary calcium (e.g. mitral annulus, pulmonary arteries). In this study, we adopted Heart-labelling method as one of the frameworks, which greatly improved the accuracy of AI prediction. The present Heart-labelling method can understand the underlying anatomy of heart using our novel training technique of using not only sparse labels but also anatomical labels. The latter one is developed for more accurate elimination of non-coronary calcium. In general, complex deep-learning models are black boxes that cannot explain the process how it judges and elicits the final output. This is considered one of the problems of current AI trends, however, our Heart-labelling method reasonably overcomes the issue.

**Figure 7:**
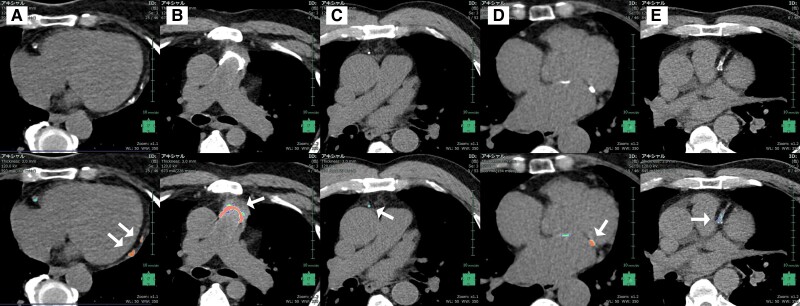
Drawbacks. There are several failure cases of the present AI-model, false positives of; (*A*) pericardial calcification, (*B*) pulmonary trunk, (*C*) calcified mediastinal lymph node, and (*D*) mitral annulus calcification, and false negative of (*E*) proximal LAD.

Although we found excellent performance of AI-derived CAC scoring in test cohort, some drawback cases should be mentioned (*Figure 7*). There were still several false positives due to misrecognition of non-coronary calcium, such as (a) pericardial calcification, (b) pulmonary trunk, (c) calcified mediastinal lymph node, and (d) mitral annulus calcification. We consider that model training was not enough to cover these kinds of cases, which is not frequent in clinical practice. (e) was a unique false negative case in which CAC in the proximal LAD was not detected by the model. It is presumably because the extraordinary LAD course, which initially descends in a caudal direction and then ascends cranial direction, was not learned sufficiently in the model training. In terms of high sensitivity of CAC scoring, false positives are acceptable rather than false negatives. We consider that in order to get over these shortcomings, more numbers of varied cases should be integrated into model training.

This study has some important limitations. First, detailed conditions for acquiring CCT images in the test cohort are unclear. Future studies need to enrol various cases with different vendors and conditions. Secondly, because the test cohort did not include calcium-absent patients, the accuracy of AI prediction may be overestimated. However, even in validation cohort including 139 (34.0%) calcium-absent cases, perfect sensitivity and NPV were found, which means it can provide robust performance in terms of screening test in clinical practice. Related to this issue, we should remark that we only validated per-patient sensitivity, not per-lesion sensitivity. Thirdly, the exclusion criteria for the images with poor quality or artefacts can be inconsistent. The manual readers determined to get rid of each image which was not to be analysed properly, and a total of 71 images were excluded. Finally, there are no consistent rules or post-processing methods for the AI to correctly segment calcifications that span multiple vascular beds. In particular, the segmentation of CAC at LM bifurcation may be clinically important, thus further refinement is needed in future studies.

In conclusion, the present Heart-labelling method provides a further improvement in fully automated, total and vessel-specific CAC detection on gated CCT.

## Supplementary Material

oead113_Supplementary_Data

## Data Availability

Most of the data underlying this article cannot be shared publicly due to the privacy of individuals that participated in the study. The data will be shared on reasonable request to the corresponding author. However, one portion of the datasets analysed in the current study is available in Center for Artificial Intelligence in Medicine & Imaging, at https://stanfordaimi.azurewebsites.net/datasets/e8ca74dc-8dd4-4340-815a-60b41f6cb2aa.

## Lead author biography

Dr. Daigo Takahashi, MD, PhD, is a cardiologist working at Juntendo University Hospital, Tokyo, Japan. He completed his medical training at Juntendo University Shizuoka Hospital. Through the career in acute cardiovascular care, he found great value in less invasive diagnostic methods, and began dedicating himself to research using cardiovascular imaging, especially in cardiac computed tomography using artificial intelligence.
