# Supplementary material for: Fully automated coronary artery calcium quantification on electrocardiogram-gated non-contrast cardiac computed tomography using deep-learning with novel Heart-labelling method
Source: Eur Heart J Open. 2023 Nov 8;3(6):oead113. doi: 10.1093/ehjopen/oead113 (PMC10683040; doi:10.1093/ehjopen/oead113)
Supplement: oead113_Supplementary_Data [file oead113_supplementary_data.docx]

**Supplemental file 1.**

**Multi-class dice loss function**

For our segmentation task, we adopted a multi-class dice loss (DL), an extension of the conventional single-class dice loss [1]. The loss function is expressed as:

$$\mathrm{DL}=\frac{1}{6}\sum_{l=1}^{6} 1-\frac{2*\sum_{n} g_{\ln}p_{\ln}}{\sum_{n} g_{\ln}+ p_{\ln}}$$

Here, $g_{\ln}$ and $p_{\ln}$ are the ground truth and prediction probabilities of $n^{\mathrm{th}}$ voxel belonging to class $l\in\{1,2,3,4,5,6\}$. This loss formulation equal weight to each class and solves the issue of class imbalance. For real images dataset, an additional weak loss $\mathrm{DL}_{\mathrm{weak}}=0.2*(1-\sum_{n} 2*g_{n}p_{n}/\sum_{n} g_{n}+ p_{n})$ is computed for the non-coronary artery region and added to the dice loss $\mathrm{DL}$. Here 0.2 is the weighting factor to reduce the impact of error in ground truth weak label. For the calculated loss, the neural network weights are updated using Stochastic Optimization method called Adam [2] with base learning rate of 0.001, beta1 & beta 2 values of 0.9, 0.999, respectively.

**Data Augmentation**

The intensities of image voxels were standardized to fall within a range of 0 to 1. We isotopically rescaled the 3D CT images to achieve a resolution that spans from 0.7 mm to 1.0 mm. Beyond scaling, we introduced random rotations of up to ±15 degrees on each axis and infused the images with random white noise, having a peak value of 0.1.

**References**

1. Çiçek, Özgün, et al. "3D U-Net: learning dense volumetric segmentation from sparse annotation." *International conference on medical image computing and computer-assisted intervention*. Springer, Cham, 2016.

2. Kingma, Diederik P., and Jimmy Ba. "Adam: A method for stochastic optimization." *arXiv preprint arXiv:1412.6980* (2014).
